# CARDIAN: a novel computational approach for real-time end-diastolic frame detection in intravascular ultrasound using bidirectional attention networks

**DOI:** 10.3389/fcvm.2023.1250800

**Published:** 2023-10-06

**Authors:** Xingru Huang, Retesh Bajaj, Weiwei Cui, Michael J. Hendricks, Yaqi Wang, Nathan A. L. Yap, Anantharaman Ramasamy, Soe Maung, Murat Cap, Huiyu Zhou, Ryo Torii, Jouke Dijkstra, Christos V. Bourantas, Qianni Zhang

**Affiliations:** ^1^ School of Electronic Engineering and Computer Science, Queen Mary University of London, London, United Kingdom; ^2^ School of Communication Engineering, Hangzhou Dianzi University, Hangzhou, China; ^3^ Department of Cardiology, Barts Heart Centre, Barts Health NHS Trust, London, United Kingdom; ^4^ Centre for Cardiovascular Medicine and Devices, William Harvey Research Institute, Queen Mary University of London, London, United Kingdom; ^5^ InfraReDx, Inc., Burlington, MA, United States; ^6^ College of Media Engineering, Zhejiang University of Media and Communications, Hangzhou, China; ^7^ School of Computing and Mathematical Sciences, University of Leicester, Leicester, United Kingdom; ^8^ Department of Mechanical Engineering, University College London, London, United Kingdom; ^9^ Leiden University Medical Center, Leiden, Netherlands

**Keywords:** end-diastolic frame, keyframe detection, recurrent neural network, intravascular ultrasound, electrocardiogram gating, medical imaging

## Abstract

**Introduction:**

Changes in coronary artery luminal dimensions during the cardiac cycle can impact the accurate quantification of volumetric analyses in intravascular ultrasound (IVUS) image studies. Accurate ED-frame detection is pivotal for guiding interventional decisions, optimizing therapeutic interventions, and ensuring standardized volumetric analysis in research studies. Images acquired at different phases of the cardiac cycle may also lead to inaccurate quantification of atheroma volume due to the longitudinal motion of the catheter in relation to the vessel. As IVUS images are acquired throughout the cardiac cycle, end-diastolic frames are typically identified retrospectively by human analysts to minimize motion artefacts and enable more accurate and reproducible volumetric analysis.

**Methods:**

In this paper, a novel neural network-based approach for accurate end-diastolic frame detection in IVUS sequences is proposed, trained using electrocardiogram (ECG) signals acquired synchronously during IVUS acquisition. The framework integrates dedicated motion encoders and a bidirectional attention recurrent network (BARNet) with a temporal difference encoder to extract frame-by-frame motion features corresponding to the phases of the cardiac cycle. In addition, a spatiotemporal rotation encoder is included to capture the IVUS catheter's rotational movement with respect to the coronary artery.

**Results:**

With a prediction tolerance range of 66.7 ms, the proposed approach was able to find 71.9%, 67.8%, and 69.9% of end-diastolic frames in the left anterior descending, left circumflex and right coronary arteries, respectively, when tested against ECG estimations. When the result was compared with two expert analysts’ estimation, the approach achieved a superior performance.

**Discussion:**

These findings indicate that the developed methodology is accurate and fully reproducible and therefore it should be preferred over experts for end-diastolic frame detection in IVUS sequences.

## Introduction

**Figure 1:**
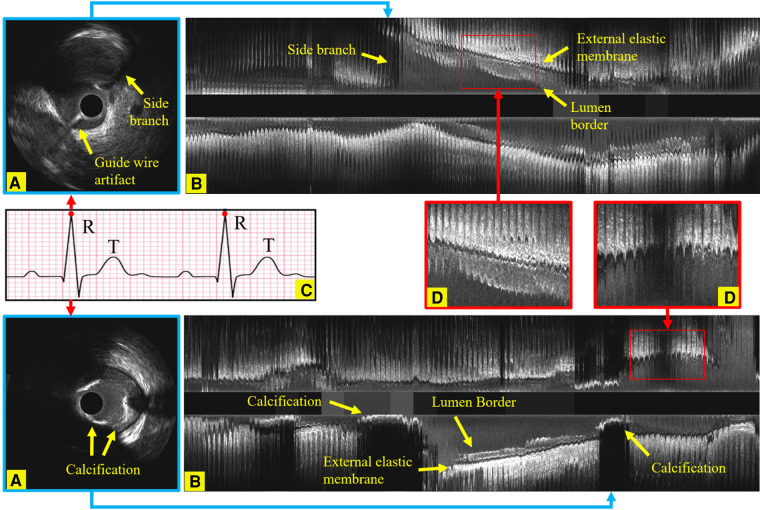
Longitudinal examples of an IVUS sequence, showing the side branches, the guide wire artifact and calcification, which all increase the challenges in identifying ED-frames. (**A**) An ED-frame. (**B**) The longitudinal view image of an IVUS pullback (3,000 frames). (**C**) An ECG signal. (**D**) Two enlarged longitudinal view images demonstrating the saw-tooth artery border caused by the movement of the vessel during the cardiac cycle.

Intravascular ultrasound (IVUS) is the preferred modality to accurately assess lumen dimensions and coronary atheroma burden in clinical practice and in research studies, playing a pivotal role in diagnosing, treating, and monitoring coronary artery disease (CAD). In contemporary practice, IVUS image acquisition is performed using an automated pull-back device that withdraws the catheter at a constant speed without gating. However, the dynamic changes in luminal dimensions during the cardiac cycle can introduce significant variability, affecting the accuracy of volumetric analysis (1). Moreover, the IVUS catheter's movement in relation to the vessel during the cardiac cycle introduces additional errors in the quantification of atheroma volume (2). Recent reports have highlighted the superiority of IVUS volumetric analysis performed in end-diastolic (ED) frames, where cardiac motion is minimized, in providing more consistent and reproducible assessments of atheroma volume. Yet, the accurate detection of these ED-frames remains a challenge due to the intricate motion of the epicardial coronary arteries and the simultaneous motion of the IVUS catheter (3). Compounding this challenge are factors like noise, artifacts, and the complex imaging environment, which further hinder the correct identification of ED frames (4), as exemplified in Figure 1. Notably, even trained experts, despite their extensive experience, often struggle to consistently identify the ED-frames. Given these challenges, there's a pressing need for a fully automated, accurate, and reproducible method for ED-frame detection, which holds the promise of revolutionizing CAD management and treatment outcomes.

Neural networks have recently been proposed for the analysis of sequential data series. The long short-term memory (LSTM)-based method has been developed for processing sequential musical audio data (5) and the detection of deception from gaze and speech (6). Recently, transformer-based methodologies were adapted for temporal information processing in natural language (7), audio (8), image (9), and video processing (10). However, these approaches require training on analyzed datasets, meaning they have limited generalizability to the ED-frame detection problem for which accurate manual labelling is unavailable.

Key frame detection in computational image analysis has been attempted with various approaches including neural networks (11, 12), clustering algorithms (13) and bidirectional LSTM (14, 15, 22). Moreover, video action recognition with skeleton-based and video-based methods has been also used for this purpose (16, 17). However, these methods contain complex encoder structures, and thus need to be trained on even larger datasets. This constraint prevents them from being readily appliable to IVUS sequences which have challenging image qualities and motion patterns. In addition, the amount of time it takes for the above approaches to process an IVUS sequence is prohibitive, making them unsuitable for clinical applications of automated IVUS analysis.

Human expert analysts tend to capture sudden changes in motion patterns when identifying ED-frames—such as reverse rotation of blood vessels and sudden start or stop of the vessel—with the presumption that the period before the largest movement of the vessel corresponds to end-diastole. Existing computational approaches for IVUS gating are based on similar assumptions and can be broadly divided into two categories: feature extraction and supervised methods. Feature extracting methods extract motion signals from IVUS pullback sequences, and gate them by identifying local extrema in the entire sequence. Since automatic ED-frame detection requires extracting key features from relative vessel motions, the main innovation has previously been to focus on exploiting motion features from shallow-learned feature representation. Several methodologies have been introduced over the recent years for IVUS ED-frame detection that relies on feature extraction including local mean intensity-based (18–20), cross-correlation based (19, 21, 22), longitudinal displacement based (21, 23–25), clustering-based (26), filter-based (3), and wavelet transform-based algorithms (27). The supervised ED-frame detection methods can be further divided into two groups: electrocardiogram (ECG)-guided methods and expert annotation-guided methods. Most current ED-frame detection methods based on ultrasound images are guided by expert annotations, meaning they use expert annotations as the gold standard (3, 27, 28). In contrast, many traditional shallow learning-based algorithms are available to solve ED-frame detection in IVUS with the support of ECG gating, such as Darvishi et al. (29), Zolgharni et al. (30), Gatta et al. (23), Isguder et al. (26), and Hernandez-Sabate et al. (18). These ECG-guided methods use simultaneously captured ECG signal to train the machine learning models (31, 32). This paper focuses on a Deep Learning-based IVUS gating approach, which distinguishes itself by employing deep learning techniques for gating, departing from the reliance on image features and signal processing for identifying key frames.

In the realm of IVUS gating, traditional methods such as ECG-based gating have been limited by synchronization challenges and susceptibility to arrhythmias. Image-based gating, although simpler, often compromises on accuracy due to the inherent complexities of images. Deep learning-based gating, as exemplified by our prior work (33), employed recurrent neural networks (RNNs) and served as a foundational step in liberating IVUS gating from ECG synchronization, thereby enhancing resilience to noise and improving accuracy. However, the current study introduces CARDIAN, a more advanced computational framework for real-time ED-frame detection in IVUS. Unlike the previous work that primarily utilized a bidirectional gated-recurrent-unit (Bi-GRU), CARDIAN incorporates a more complex BARNet to exploit both forward and backward motion features in IVUS sequences. It also employs meticulously designed high-performance encoders—Temporal Difference and Spatiotemporal Rotation—for robust feature extraction. The framework is further enriched by a dual-layer Bidirectional Long Short-Term Memory (Bi-LSTM) structure with attention mechanisms, allowing for the processing of longer input sequences and offering more accurate post-processing. Rigorous training and testing protocols, including leave-one-out and three-fold cross-validation methods, are outlined. Additionally, novel strategies for unit acquisition and data augmentation have been introduced to adapt the model to various vessel wall motions and other artifacts. Developed in partnership with industry (InfraReDx, Inc., Burlington, Massachusetts), CARDIAN has the potential to be incorporated into commercially available systems for real-time processing of near-infrared spectroscopy-IVUS images. This multi-faceted approach significantly extends the scope, robustness, and versatility of our previous work, aiming to set a new standard in the accuracy, efficiency, and reliability of IVUS ED-frame detection. To substantiate the efficacy of CARDIAN, we have conducted rigorous internal validation using three-fold cross-validation methods. Furthermore, we have benchmarked our approach against Image-based gating methods, which are widely employed in commercial IVUS analysis software, thereby providing a comparative perspective on its performance.

The main contributions are summarized as follows:

- CARDIAN is proposed, namely, a novel Computational Approach for Real-time end-diastolic frame Detection in Intravascular ultrasound (IVUS) using bidirectional Attention Networks. CARDIAN utilizes a bidirectional recurrent neural network (BARNet) to exploit the forward and backward motion features in IVUS sequences with the guidance of a temporal attention scheme trained on gold standard data obtained by ECG gating.
- A framework based on CARDIAN is implemented for ED-frame detection in IVUS sequences, which includes an IVUS-sequence denoising and motion encoding module, a BARNet network for predicting the likelihood of a frame being an ED-frame, and an ED-frame search module for accurate identification of ED-frames based on a generated probability graph.
- Demonstration of the superior performance of the CARDIAN methodology compared to human expert analysts and conventional motion feature-based ED-frame gating methodologies. CARDIAN shows promising results in accurately detecting ED-frames, even in challenging scenarios with noise, artifacts, and complex imaging environments.
- Evaluation of the CARDIAN methodology using metrics such as group-of-pictures (GoP) recall, GoP precision, GoP F1 score, and nearest prediction interval (NPI), which provide insights into its effectiveness in identifying ED-frames with high detection rates and minimized errors.
- Validation of the CARDIAN methodology in NIRS-IVUS sequences, showing its robust performance across different coronary arteries and its potential for clinical and research applications in coronary artery disease (CAD) management.

In this study, we provide a detailed description of the proposed method in Chapter 2. The performance of the proposed method is evaluated and analysed in practical scenarios, and compared against the state-of-the-art methods in Chapter 3. The discussion in Chapter 4 provides further insights into the effectiveness of the proposed method, and its potential for clinical applications is analysed.

## Materials and methods

**Figure 2:**
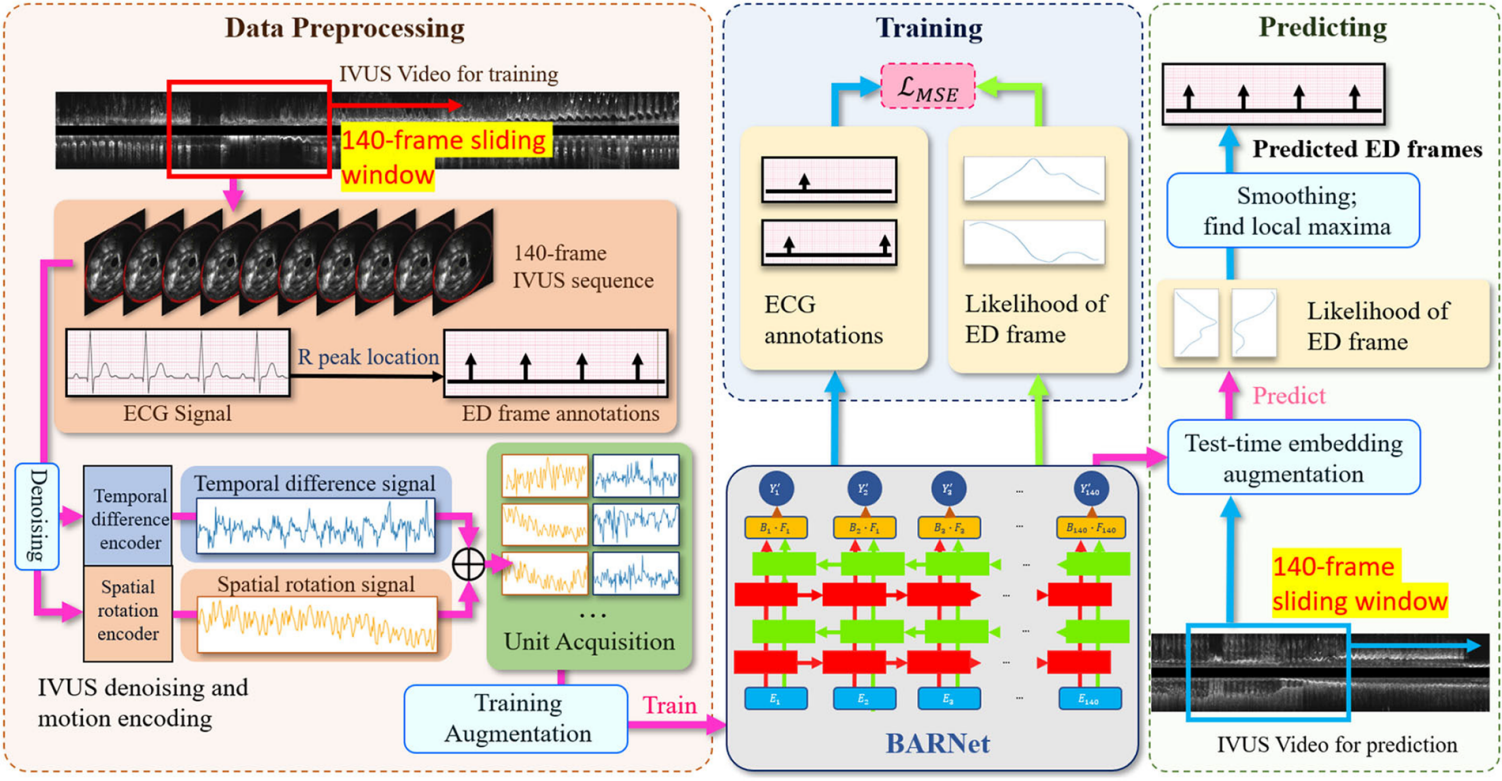
The overall architecture of the proposed CARDIAN methodology, depicting the consecutive stages from data processing to ED-frame detection. The process commences with using a 140-frame acquisition window to extract relevant segments, followed by identifying ED-frames using ECG signals. Next, feature signal extraction is performed to gather relevant motion information. Data augmentation is then applied to improve model adaptability and accuracy. Then, BARNet is employed to predict the likelihood of a frame being an ED-frame in each unit. Finally, the ED-frame search module arranges the units sequentially and further explores the accurate positions of ED-frames based on a generated probability graph.

The overall architecture of the proposed CARDIAN methodology is illustrated in Figure 2. In the following, every component in this architecture is introduced in detail.

### Data acquisition

In the data acquisition process, this study encompassed six participants diagnosed with obstructive coronary artery disease who were undergoing coronary angiography and percutaneous coronary intervention. These individuals were enlisted in the “Evaluation of the effectiveness of computed tomographic coronary angiography (CTCA) in the evaluation of coronary artery morphology and physiology” investigation (NCT03556644), forming the basis of the current analysis. The core procedure involved subjecting all patients to near-infrared spectroscopy intravascular ultrasound (NIRS-IVUS) imaging of their coronary arteries and significant lateral branches. This imaging was conducted using the innovative Dualpro NIRS-IVUS system, developed by Infraredx, located in Burlington, MA.

To orchestrate the process meticulously, the NIRS-IVUS probe was systematically retracted at a uniform pace of 0.5 mm/s, facilitated by an automated pull-back apparatus. Concurrently, data acquisition occurred at a rate of 30 frames per second (fps). While this transpired, an electrocardiogram (ECG) trace was concurrently recorded alongside the IVUS sequence. Notably, the frame rate for this ECG data was elevated to 120 fps. A visual representation of this synchronization and coordination can be observed in Figure 1, where the IVUS sequence and ECG trace converged, allowing for seamless co-registration and precise identification of the IVUS frame that corresponded to the zenith of the R-wave, an event termed the ED-frame.

The 50 MHz Dualpro system developed by Infraredx in Burlington, Massachusetts was employed for NIRS-IVUS imaging. Localization of the NIRS-IVUS probe was achieved through the introduction of a contrast agent, which was succeeded by the acquisition of an angiographic projection following the administration of 400mcg of nitrates. This preparatory phase facilitated the subsequent advancement of the NIRS-IVUS probe towards the distal section of the vessel.

During the pullback procedure, precision was maintained through the use of an automated pullback device, ensuring a consistent velocity of 0.5 mm/s. This pullback action was concomitantly accompanied by the capture of an ECG trace. The NIRS-IVUS pullback process was meticulously synchronized with the ECG data. A frame rate of 30 fps was allocated to the automated pullback mechanism, while a camera equipped with a heightened frame rate of 120 fps was deployed to record the amalgamated display, showcasing both the NIRS-IVUS data and the ECG trace.

A manual inspection was then administered by experts in the field. They engaged in a comprehensive review of the amalgamated video footage, which harmonized NIRS-IVUS data and ECG signals. This meticulous analysis involved the manual annotation of all end-diastolic frames within the NIRS-IVUS recording, guided by the cues provided by the ECG signals. The identification of the IVUS frame corresponds to the peak of the *R*-wave within the ECG signal, which was aptly documented as the end-diastolic frame and gold stranded of the research. For the purpose of rigorous validation, the dataset was carefully partitioned into three folds, adhering to a patient-based stratification approach. This ensured that pullbacks from the same patient were not present in both the training and test sets within each fold. Additionally, efforts were made to balance the number of ED-frames across these folds to maintain a consistent level of challenge for the model during the cross-validation process.

### Frame denoising and motion encoding

The conventional encoders in CNN mainly focus on pixel-level short-term relationships (34, 35). We observed that the most relevant information for identifying ED-frames in IVUS pullbacks is the relative motion of the coronary arteries with regards to the IVUS probe. Thus, we aim to design encoders that can extract dynamic change data across frames as descriptive features. The IVUS images contain significant noise that can interfere with the encoding of key information. It is essential to smooth this noise and reduce its obfuscating effects, allowing the model to focus on the periodic motion features induced by the cardiac cycle rather than fluctuations from noise, and consequently improve extraction of clinically relevant features from the IVUS imagery. To achieve this, we have implemented a guided image filter for smoothing out perturbations. The filter operates according to the equation G(x)=a⋅I(x)+b, where G(x) is the output, I(x) is the input image, and *a* and *b* are two constants. This denoising technique effectively mitigates the impact of noise, thereby allowing the model to concentrate on the cyclical motion characteristics intrinsic to cardiac activity.

After noise filtering, we developed two specialized encoders: the Temporal Difference Encoder and the Spatiotemporal Rotation Encoder, to extract motion feature caused by cardiac motion. The details of which will be elaborated upon subsequently. The encoders not only capture the dynamic changes across frames but also enhance the model's resilience to noise and other confounding factors.

### Temporal difference encoder

**Figure 3:**
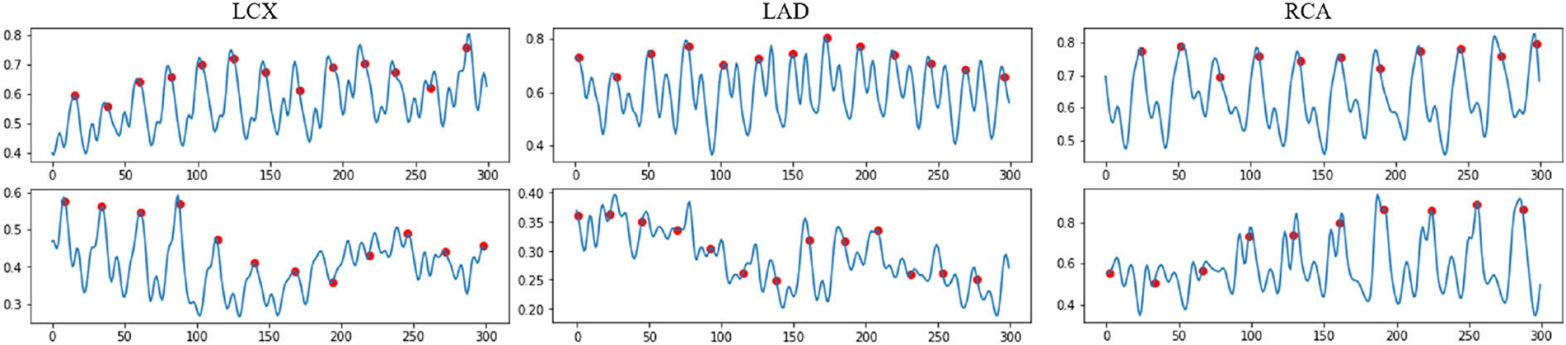
Examples of the normalized encoded temporal feature sequences, from original IVUS sequences. The blue line represents the temporal feature data, which is extracted by the proposed temporal difference encoder. The red dots represent the ED frames detected by the ECG. Each sequence includes 300 frames.

The IVUS sequences are projected onto a one-dimensional feature signal where each value represents how much difference there is between every two adjacent IVUS frames. The change data between every two frames are calculated by the sum of absolute pixel intensity differences:

(1) $$e_{n}=\overset{H}{\underset{i=1}{\sum }}\overset{W}{\underset{j=1}{\sum }}|P_{n+1}^{i,j}-P_{n}^{i,j}|,$$

where $e_{n}$ is the encoded motion feature between two consecutive frames $f_{n}$ and $f_{n+1}$ with frame resolution H×W. $P_{n}^{i,j}$ is a pixel's intensity in the frame $f_{n}$, where *i* and *j* representsthe pixel coordinates. Some examples of the temporal difference motion features are shown in Figure 3. It can be observed that most motion peaks are strongly correlated to the *R* peaks in ECG, but not always, particularly in vessels with excessive motion like the right coronary artery (RCA).

The temporal difference encoding is simple yet effective in reducing the quality demand for the input sequence, allowing the method to work on pullbacks captured by different catheters with different frame rates.

### Spatiotemporal rotation encoder

**Figure 4:**
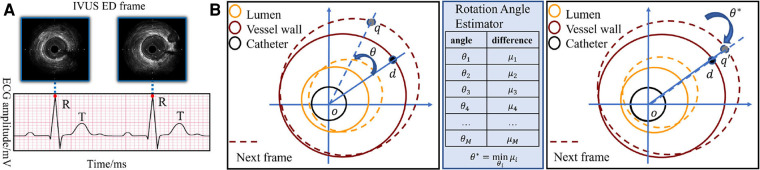
(**A**) The correspondence between the peak of R wave in ECG and ED-frames in IVUS. (**B**) Catheter rotation alignment between two consecutive frames. The spatiotemporal rotation encoder aligns two consecutive IVUS frames by rotating the vessel around the catheter center. The rotation angle θ is estimated by minimizing the difference between the adjacent rotated frames, and the aligned frames are then used for feature extraction. The pixel *d* in the first frame is rotated to the position *q* in the next frame.

The rotational motion of the vessel during the cardiac cycle is key information for ED-frame feature extraction. Therefore, a spatiotemporal rotation encoder is designed to extract the vessel's rotation features. First, a rotation angle extractor (RAE) is developed to align two consecutive frames. Figure 4B illustrates the rotation of the vessel around the catheter center *o* between two adjacent IVUS frames. Assuming a rotation angle θ the pixel *d* is rotated to the position *q* in the next frame. To estimate the actual rotation angle θ, we first determine the angle range based on the prior information of the acquisition device. Then we obtain a set of angles $\theta_{x}$ by averagely sampling all angles in this range, where *x* is the angle index. We denote the rotation operation around the center of the catheter as R. The estimated angle $\theta^{*}$ is calculated by minimizing the difference between adjacent rotated frames by Equation 2 and Equation 3. In Figure 4A, the catheter in $f_{n+1}$ is rotated by $\theta^{*}$ degrees clockwise to align the two adjacent frames. After that, the pixel *d* in the current frame, and q′ in the next frame are on the same line. We apply the spatiotemporal rotation encoding frame by frame in each IVUS sequence.


(2) $$\mu_{x}=\overset{H}{\underset{i=1}{\sum }}\overset{W}{\underset{j=1}{\sum }}|R_{\theta_{x}}(P_{n+1}^{i,j})-P_{n}^{i,j}|,\;$$

(3) $$\theta^{*}=argmin_{\theta_{x}}(\mu_{x}).\;$$

The artery motion captured by spatiotemporal rotation encoding is calculated for each two adjacent and aligned IVUS frames:

(4) $$e_{n}^{\prime }=\overset{N}{\underset{i=1}{\sum }}\overset{M}{\underset{j=1}{\sum }}|R_{\theta^{*}}(P_{n+1}^{i,j})-P_{n}^{i,j}|.\;$$

**Figure 5:**
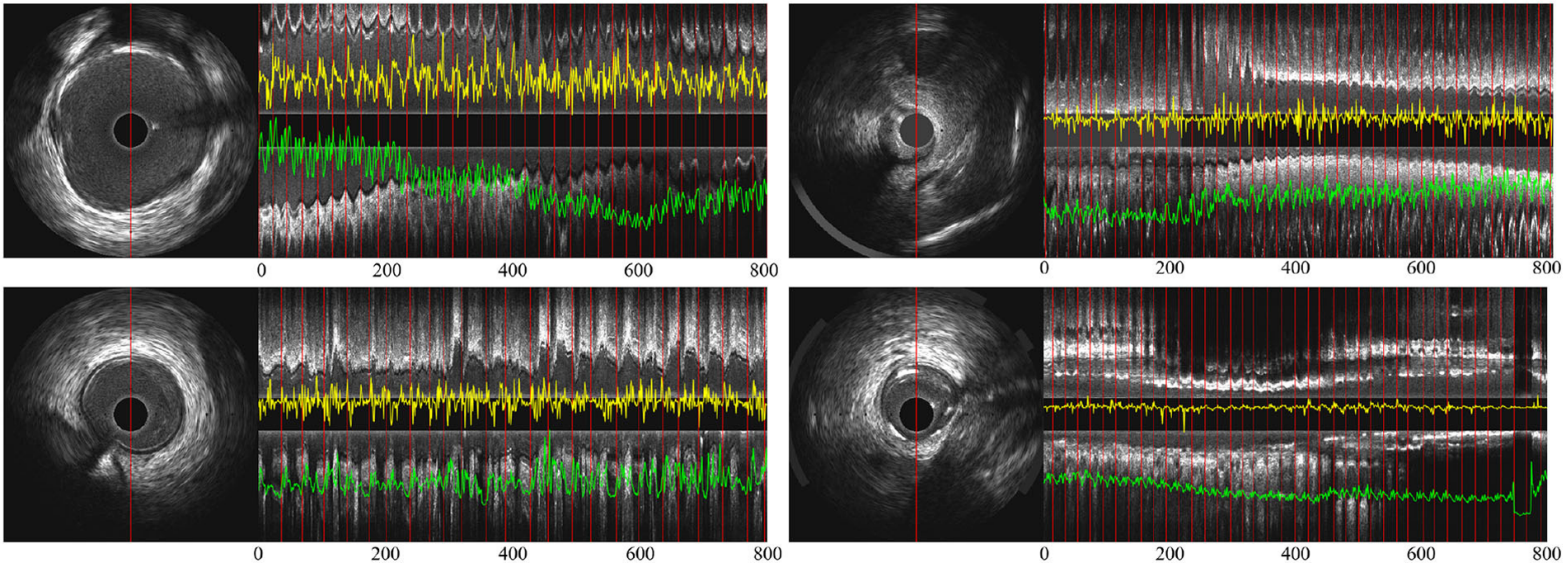
(**A**) Partial longitudinal views and the proposed encoded sequence in 4 IVUS sequences—the length of each partial longitudinal view is 800 frames (26.6 s). The image on the left is a representative IVUS frame with the red line showing the sampled pixels used in the partial longitudinal view ($0^{\circ }$). The red lines in the longitudinal view indicate the ED-frames annotated by ECG. The yellow curves show the feature sequence encoded by the spatiotemporal rotation encoder and the green the temporal difference encoded sequence.

Figure 5 shows the signals encoded by the temporal difference encoder and spatiotemporal rotation encoder, together with the corresponding longitudinal view for the original IVUS pullback. In the first three cases, the encoded signals from intense and regular cardiac motion show a clear cycle of motion between ED-frames, demonstrating the regular systolic relaxation of the heart. In the last case on the lower right, the movement of the vessel is small, and thus the coded movement feature is weak as well as vessel's rotation. The proposed temporal difference encoder and spatiotemporal rotation encoder transfers 2D image signal to 1D global temporal features to reduce the demand for GPU performance for ED-frame detection and eliminates the interference factors during data collection such as noise, rotation, and local diseases of vessels.

### Training the CARDIAN

In the training stage, first, the input IVUS frames are de-noised to minimize the influence of imaging artifacts. Then, the frames are encoded by the two lightweight encoders, the temporal difference encoder and the spatiotemporal rotation encoder, to generate a descriptive representation of the IVUS sequence in the temporal domain. The representation is then reorganized into small units which are used to train the BARNet model together with the reference ECG-derived ED-frame as the gold standard. Through the training, BARNet learns to predict the likelihood of each frame being an ED-frame.

### Testing the CARDIAN

The trained model is then tested using the leave-one-out cross-validation approach—all sequences are used for training apart from the sequence of one vessel. This is done for each vessel type, namely, left anterior descending (LAD), left circumflex (LCx) and right coronary artery (RCA). This process is repeated leaving a different vessel out from the training set each time until all vessels are used for testing.

A three-fold cross-validation method was applied to the matched ECG-IVUS data to compare the performance of experts and automated methodologies. In each fold, the dataset was evenly distributed among the three types of coronary arteries: RCA, LAD, and LCx. To tackle the data imbalance problem, the underrepresented vessel type frames were multiplied to ensure an equivalent representation for each type. To mitigate the risk of data leakage, each pull-back was strictly allocated to either the training or testing set within a given fold, ensuring no overlap between the two sets. Specifically, in each fold, approximately 67% of the pull-backs were designated for training, while the remaining 33% were exclusively used for testing. The performance metrics were then averaged across all folds to provide a robust estimate of the overall efficacy of the proposed method. We then averaged the results to estimate the overall performance of the proposed method.

### Unit acquisition

**Figure 6:**
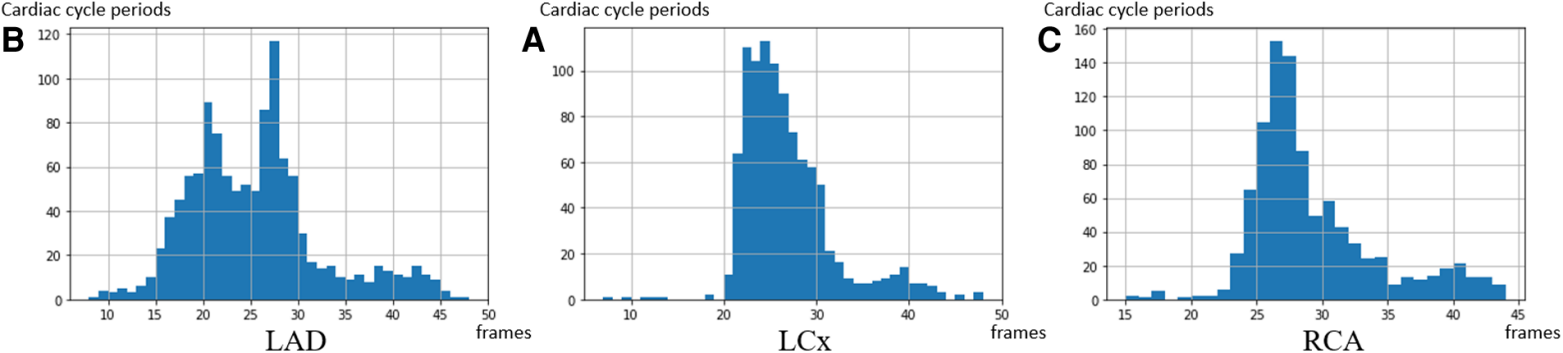
The distribution of the cardiac cycle period in the 3 coronary arteries. The average heart rate in the dataset is 67 heartbeats per minute—corresponding to a 27 frames interval per heartbeat. (**A**) LAD, (**B**) LCx, and (**C**) RCA.

The distributions of cardiac cycle durations in three types of arteries are shown in Figure 6. Based on our dataset, the average cardiac cycle is about 900 ms or 27 frames. To prepare suitable inputs to the detection model, a sliding window of 140-frame length is applied to the encoded motion feature of every pullback with a step size of 1. The 140-frame window roughly covers the motion of five cardiac cycles. This means that for each IVUS sequence with *K*, a total number of frames K−139 encoded motion segments $\left\{E_{1},\;E_{2},\;.\;.\;.,\;E_{k-139}\right\}$ are acquired, where $En\;=\;\{e_{n}^{1},\;e_{n}^{2},\;.\;.\;.,\;e_{n}^{140}\},\;n\;=\;1,\;2,\ldots ,\;K\;-\;139$. Each corresponding ECG signal goes through the same process to obtain matching ground truth units $\left\{Y_{1},\;Y_{2},\;.\;.\;.,\;Y_{k-139}\right\}$. After that, pairs of encoded IVUS feature units and ECG signal units covering a 140-frame length are prepared as the training input.

The units of 140 frames, which roughly cover five cardiac cycles can provide a wider view of the network and reduce the chance of confusing T peaks with R peaks. An ED-frame lies around the middle between every two cardiac cycles. This unit setting allows our model to determine the locations of ED-frames based on the temporal motion information over a longer term. In addition, among the multiple ED-frames, each one can use the others as references in the prediction process.

### Augmentation

Data augmentation serves as a critical step in our pipeline, performed prior to feature extraction. The primary objective is to equip the model with the ability to generalize across a wider range of vessel wall motions, cardiac cycle amplitudes, and other IVUS-specific artifacts such as plaque morphology, ventricular or atrial ectopics, and random noise. This strategy aims to mitigate the impact of these variables on detection accuracy.

Our augmentation techniques include random interpolation, frame elimination, and the addition of Gaussian noise. Specifically, for each 140-frame unit, we randomly remove 1–5 frames and replace them with new synthetic frames, the values of which are computed as the average of adjacent frames. This stochastic alteration of the IVUS sequence effectively modulates the cardiac cycle period, thereby training the model to adapt to varying heart rates. Furthermore, we introduce a random scaling factor between 0.8 and 1.2 to each frame's pixel values and add a 10% Gaussian noise to the feature signal. These steps are designed to make the model resilient against IVUS artifacts and improve its ability to discern genuine vessel and lumen characteristics.

While generative models offer the potential for creating synthetic inputs, they often require a large volume of training data to produce reliable and highly resembling outputs. Given the specialized and complex nature of IVUS imaging, and the challenges associated with data collection, a poorly trained generative model could introduce more noise and confounding variables, thereby potentially degrading the model's performance. Therefore, we opted for targeted, clinically explainable augmentation techniques that are specifically tailored to address the unique challenges of IVUS imaging. By employing these augmentation techniques, we aim to create a more versatile and robust training set, thereby enhancing the model's performance and generalizability.

### Bidirectional attention recurrent network

**Figure 7:**
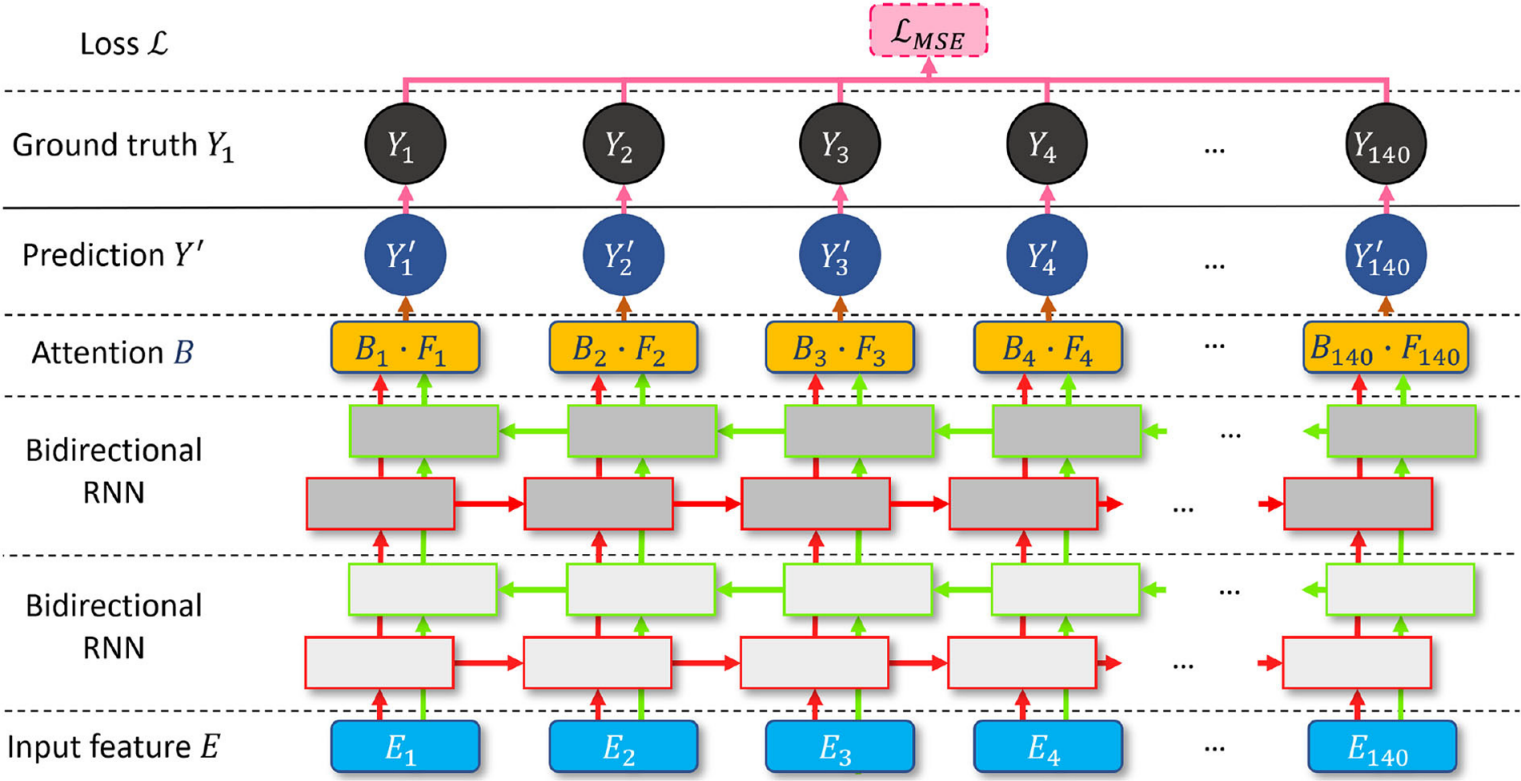
A schematic illustration of the proposed bidirectional attention recurrent network (BARNet) for ED-frame detection. The network consists of two bidirectional RNN layers (GRU or LSTM), followed by an attention layer to learn long-term dependencies, and finally generating the predicted ED-frame likelihoods. The encoded IVUS features are input into the network, and the output is the probability of each frame being an ED-frame.

RNN (36) and attention (37) have become milestone techniques for text classification and speech recognition tasks. Recently, the transformer has become one of the most popular state-of-the-art attention branches (38). Inspired by its robust performance, we design a bidirectional attention recurrent network (BARNet) to detect ED-frames on the encoded IVUS features, as depicted in Figure 7. We apply two bidirectional gated recurrent units (GRUs) (39) or LSTM (40) as the first two layers of BARNet. The reason for considering a bidirectional RNN (41) is that the relevant motion features involve adjacent frames both before and after the target position. The output features of the bidirectional RNN pass through an attention layer to further learn the long-term dependency inside each unit, as shown in the BARNet block in Figure 2. For a long sequence with 140 cells, some intermediate state information will inevitably be lost in the middle cells. Compared with bidirectional LSTM and GRU, the attention layer in BARNet has a higher reception field for better learning the long-term dependencies. This capability has obvious advantages in ED-frame detection, by giving higher weights to frames with larger motion amplitude and focusing on these key frames for generating predictions.

We input the encoded feature units $E_{n}$ into the two-layer bidirectional RNN and denote the output of the layer as $F_{n}\in \;R^{140\times 1}$. Then the attention weight $A_{n}\in \;R^{140\times 1}$ of the feature unit $E_{n}$ is calculated based on a normalized element-wise multiplication with the learnable weight $W\in \;R^{140\times 1}$, as shown in Equation 5:

(5) $$A_{n}=\frac{W\circ F_{n}}{\sum_{t}(W_{t}F_{n,t})},\;B_{n}\;=\;exp(tanh(A_{n}));\;$$

where $W_{t}F_{n,t}$ is the influence of the $n^{th}$ unit on the $t^{th}$ feature element of the target. To bring in more non-linear information and increase the margin between ED-frames and non-ED-frames, we obtain the local attention $B_{n}$ using a *tanh* function and an exponential *exp* on the normalized $A_{n}$. The exponential function is used to alleviate the gradient vanishing problem of the *tanh* operation. The predicted ED-frame likelihood $Y_{n}^{\prime }\in \;R^{140\times 1}$ of each element n is generated by the element-wise multiplication between the local attention $B_{n}$ and the encoding $F_{n}$, as shown in Equation 6:

(6) $$Y_{n}^{\prime }\;=B_{n}F_{n},\;n\in \{1,2,\ldots ,140\}.$$

The ground truth on ED-frames is denoted as $Y_{n}\;=\{y_{n}^{1},y_{n}^{2},\ldots ,y_{n}^{140}\},\;y\in \;\{0,\;1\}$. $y_{n}\;=\;1$ when the $n^{th}$ frame is an ED-frame. A BARNet model is then trained using encoded motion segments $E_{n}$ and their corresponding ground truth ED-frames $Y_{n}$. The ED-frame likelihood $Y_{n}^{\prime }$ of the motion segment $E_{n}$ is predicted by minimizing the mean squared error (MSE) loss $L_{MSE}$ in every training epoch (42).

### ED-frame search module

In the proposed method, the spatiotemporal rotation feature and temporal difference feature are used as inputs to generate more robust prediction results. Inspired by the argumentation methods in image classification and segmentation tasks, we proposed a test-time embedding augmentation (TTEA) scheme for IVUS ED-frame detection. In the prediction stage, for a unit of 140 frames $\left\{E_{1},\;E_{2},\;.\;.\;.,\;E_{k-139}\right\}$, each frame is randomly multiplied by a number from 0.8 to 1.2 and added with a 10% Gaussian noise. The model will generate a prediction on this version of the unit. The augmentation and prediction processes are repeated 50 times, and the output of 50 probability graphs is averaged to obtain the final probability graph, indicating the likelihood of each of the 140 frames being an ED-frame $\left\{y_{1}^{\prime },y_{2}^{\prime },\ldots ,y_{K-139}^{\prime }\right\}$. The mean likelihood value *v* for each frame is considered as the final likelihood of the corresponding frame being an ED-frame. Since the duration of an average cardiac cycle is equivalent to 27 frames, a Hanning smoothing window of size 13 is performed on the final probability graph *v*. To avoid identifying more than one ED-frame in a cardiac cycle, a 13-frame sliding window will go through the smoothed probability graph, and the local maxima on the $7^{th}$ frame of a sliding window will be finally identified as an ED-frame.

### Performance evaluation

The performance of the CARDIAN was compared with the visual screening results of two expert analysts from an intravascular imaging core-lab. They reviewed the IVUS pull-backs and identified the end-diastolic frames as the frame with the minimum vessel motion before a sudden motion of the vessel in relation to the catheter. Furthermore, we compared the performance of CARDIAN with an automated ED-frame detection methodology for retrospective gating of IVUS images. This automatic method relies on detecting neighboring frames where the lumen motion is minimal (LM-method) (43). This methodology has been incorporated in a user-friendly software, the QCU-CMS IVUS image analysis software (Leiden University Medical Center, Leiden, The Netherlands), and has been extensively used in the past to identify the ED frames in clinical research.

### Statistical analysis

For quantitative evaluation in this study, numerical variables are presented as mean ± standard deviation (SD), and categorical variables as absolute values and percentages. The chi-squared test was used to compare categorical variables. Bland–Altman analysis was employed to compare the estimations of expert analysts, the conventional image-based approaches (LM) (43) and the proposed method CARDIAN.

To effectively demonstrate the performance in ED-frame detection, in this paper, we define a few new metrics, namely, group-of-pictures (GoP) recall, GoP precision and GoP F1 score. In calculating these recall/precision values, each detection is considered a hit if the predicted ED-frame is within a tolerance range of ±3 frames of the target frame, that is, ±66.7 milliseconds (ms) in time. This approach follows the evaluation paradigm used in evaluating human labelling of ED-frames in previous studies (33), but it uses a tighter range of ±number of frames or time.

The GoP recall is defined mathematically as:

$$GoP\;recall\;=\frac{\;\sum_{i=1}^{n}I\left(\left|P_{i}-R_{i}\right|\leq PT\right)}{n}\;\times \;100\%$$

In this study, the prediction tolerance (PT) is set to be a range of ±66.67 ms or 2 frames from the peak of the R-wave on the ECG, but it can be adapted to other values as appropriate. GoP recall represents the percentage of correctly detected frames ($P_{i}$) within the range of PT from their closest ED frame by ECG ($R_{i}$). The total number of ECG-derived ED-frame is denoted as ‘n’. The summation runs from i=1ton, the indicator function I(condition) is calculated at each position, which equals 1 if the absolute distance between the predicted frame ($P_{i}$) and its corresponding closest ED frame in ECG ($R_{i}$) is smaller than the specified range PT; otherwise, it equals 0.

Similarly, group of Picture (GoP) precision is defined mathematically as:

$$GoP\;precision\;=\frac{\;\sum_{i=1}^{m}I\left(\left|P_{i}-R_{i}\right|\leq PT\right)}{m}\;\times \;100\%$$

Here, ‘m’ denotes the total number of the detected frames. The summation runs from i=1 to *m*, and each indicator I(condition) is calculated, which equals 1 if the absolute distance between each frame classified as ED $\left(P_{i}\right)$ by the experts or the tested methodology and its corresponding closest ECG-derived ED frame $\left(R_{j}\right)$, is smaller than the specified range PT; otherwise, it equals 0. GoP precision represents the percentage of the correctly detected ED frames (P_i) out of the total number of the detected frames by an expert or an algorithm.

GoP F1 score is a measure that combines both GoP recall and GoP precision into a single value, providing a balanced representation of the method's performance. It can be calculated using the following formula:

$$GoP\;F1\;Score\;=\;2\;\times \frac{\left(GoP\;Recall\;\times \;GoP\;Precision\right)}{\left(GoP\;Recall\;+\;GoP\;Precision\right)}$$

The F1 score ranges from 0 to 100%, with higher values indicating a better performance in terms of high detection rate as well as minimized errors.

Additionally, nearest prediction interval (NPI) was employed to measure the average time interval between every detected ED-frame and its closest ECG-derived ED-frame. Given the total number of predictions (n) and the distance between each detected ED frame ($P_{i}$) and its closest real frame ($R_{i}$), NPI can be computed as:

$$NPI=\frac{\sum_{i=1}^{n}\left|P_{i}-R_{i}\right|}{n}$$

These metrics together offer a multi-perspective insight into the effectiveness of the automated and manual methods for ED-frame detection.

## Results

This study involved patients with an average age of 61.7 ± 10.3 years and 83.3% of them were male. None was a smoker but most of them had a positive family history of CAD (66.7%), hypertension (66.7%) and hypercholesterolemia (66.7%). Five patients (83.3%) had normal and one had impaired left ventricular function. The studied vessels (*n* = 20) included 9 LCx, 6 LADs and 5 RCAs. Out of the 92,526 frames acquired from these vessels; after excluding cases of non-interpretable IVUS images and ECG tracings because of artifacts, 3,271 were classified as ED by the ECG. The average heart rate was 66 beats per minute.

After adding segments in each vessel type with fewer ED frames to obtain a more balanced dataset—as described in the methodology section—a total of 3,556 ED-frames were included in the analysis, of which 1,269 ED-frames were located in the LAD, 1,133 in the LCx, and 1,154 in the RCA.

### Ablation study

An ablation study was performed to determine the effect of each proposed module, as reported in Table 1. The proposed training augmentation mechanism significantly improved the efficacy of the method to detect the correct ED frames. Since RNN-like structures are sensitive to the cardiac cycles, by randomly changing each cardiac cycle length, networks can better adapt to patients’ data with different cardiac cycles. Further, the noise and random disturbance added into training set reduced the influence of challenging areas like frames with large plaques, artifacts, side-branches, or noise from the catheter, helping the model to capture critical features from the input signal. The proposed TTEA module also marginally increased the performance of all experiments.

**Table 1:** Ablation study on the ECG-IVUS dataset based on GoP recall.

%		No training augmentation						Training augmentation					
Backbone		Bi-GRU			Bi-LSTM			Bi-GRU			Bi-LSTM		
TTEA		None	Tem.	Both	None	Tem.	Both	None	Tem.	Both	None	Tem.	Both
w/o att.	Fold 1	69.08	72.38	72.58	68.75	69.02	70.93	66.51	66.58	65.59	72.58	73.37	74.75
	Fold 2	63.17	64.46	63.56	63.96	62.48	64.85	59.60	62.38	63.47	64.55	65.54	66.83
	Fold 3	57.92	58.89	60.45	57.92	59.38	58.50	64.24	62.59	63.65	62.59	62.49	62.78
	All	64.17	66.23	66.51	64.26	64.37	65.61	63.89	64.23	64.43	67.41	68.00	69.04
with att.	Fold 1	71.00	72.64	71.85	70.73	71.32	70.67	72.58	73.83	73.70	70.80	69.94	72.18
	Fold 2	66.44	66.14	67.62	67.33	66.24	66.24	66.63	65.64	66.24	67.33	69.01	69.31
	Fold 3	60.54	62.49	62.10	60.16	61.03	63.46	62.20	66.96	66.67	65.60	66.67	67.25
	All	66.68	67.86	67.83	66.70	66.90	67.32	67.89	69.52	69.54	68.31	68.73	**69** **.** **94**

Test-time embedding augmentation (TTEA) is not applied (None), applied on the temporal difference encoded features (Tem.) or on both temporal difference encoded and spatiotemporal rotation encoded features (Both). The model performance with or without attention (Att.) are both evaluated. The bold value indicates the highest accurate rate in all experiments.

We also found that Bi-LSTM models outperforms Bi-GRU. Further, the attention layer provides an 1%−3% increase in both models. Since the Bi-LSTM or Bi-GRU in the experiment both have a length of 140 cells, a primary issue for such long RNN structures is that the information shared by distancing cells is very faint. In this situation, the attention layer plays an important role by providing a larger field of view to help generate more accurate predictions, eliminate false predictions caused by other movements and prevent overfitting.

**Figure 8:**
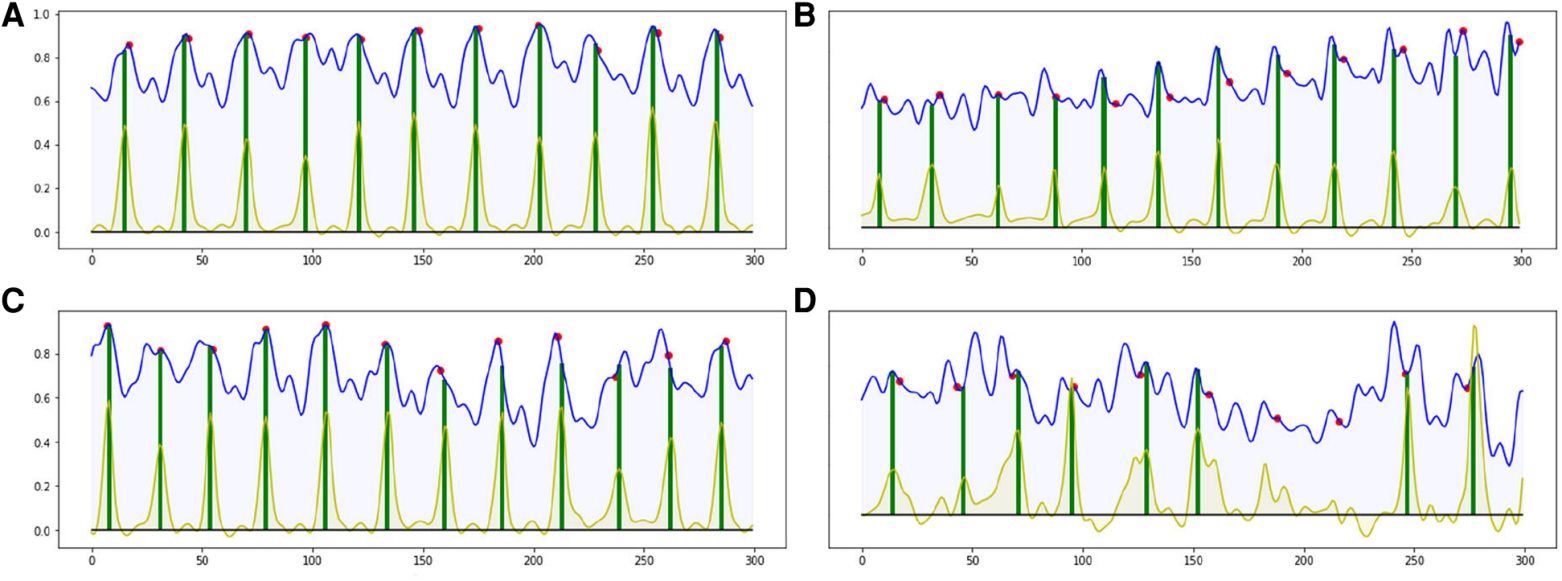
Four result samples generated by BARNet in 300 frames (10 s). Blue curves: features encoded by the temporal difference encoder; red dots: ED-frames ground truth based on ECG; yellow curves: BARNet generated prediction; green lines: final ED-frame prediction based on the prediction scores of CARDIAN. Images (**A**–**C**) show results generated on regular cardiac cycles, and (**D**) presents the result for a patient with frequent ectopics and irregular cardiac cycles.

Most of the ED-frames can be predicted correctly by our end-to-end CARDIAN approach. Figure 8 illustrates the detection results on four IVUS sequences based on the best configuration. In Figure 8A–C, with clear cardiac motion patterns, the proposed framework detects all the ED frames with a high accuracy. In an IVUS pullback with irregular cardiac movement and extensive disease (Figure 8D), the performance of the model is impaired, but most of the ED frames can still be roughly located.

The detection errors are often due to the variance in motion patterns, the accuracy of the network declines when the IVUS frames have an irregular motion or barely move. This usually occurs in sequences portraying coronary heart diseases as shown in Figure 8D.

### ED frame detection in NIRS-IVUS sequences

For quantitative evaluation, ED-frame detection results from two human analysts, a conventional image-based approach (LM), and CARDIAN are compared. As summarized in Table 2, the two analysts correctly identified 808 (22.72%) and 1,032 (29.02%) ED-frames, and missed 907, 814, and 1,027 ED-frames in LAD, LCx, and RCA, respectively (Table 2). Meanwhile, the analysts incorrectly identified 3,006 and 2,762 frames as ED-frames while these do not correspond with cardiac cycles based on the prediction tolerance of 66.7 ms. Among these, the number of falsely identified frames in the RCA was higher (Exp.1: 1,107, 89.71%; Exp2: 1,006, 81.72%) compared to LAD (Exp.1: 1,024, 73.88%; Exp2: 964, 70.06%) and LCX (Exp.1: 875, 73.28%; Exp2: 792, 66.72%).

**Table 2:** Efficacy of the expert analysts of the LM and of the CARDIAN method in detecting the ED, using a prediction tolerance of 66.7 ms (2 frames).

	ECG-defined ED frames	Expert 1	Expert 2	LM	CARDIAN
Number of frames identified as ED-frames	3,556	3,814	3,794	3,760	4,405
Predicted frames that could not be matched with the ECG estimations (False positive)	–	3,006	2,762	3,045	1,918
Missing ED-frames (False Negative)	–	2,748	2,524	2,841	1,069
Correctly classified ED-frame (True Positive)	–	808	1,032	715	2,487

The LM methodology correctly detected 715 ED-frames (20.11%), wrongly detected 3,045 (80.98%) frames and did not detect any ED-frames in 2,841 (79.89%) cardiac cycles. The numbers of false detected frames by the LM method were similar in LAD (1,086, 79.62%), LCx (965, 81.50%) and RCA (994, 82.01%).

In comparison, the proposed CARDIAN method correctly detected 2,487 (69.94%) ED-frames. To be specific, CARDIAN detected 912 (71.87%), 768 (67.78%), and 807 (69.93%) ED-frames in LAD, LCx, and RCA, respectively, and missed 357 (28.13%), 365 (32.22%), and 347 (30.07%) ED-frames in these vessels. Moreover, 690 (43.07%), 661 (46.26%), and 567 (41.27%) frames were wrongly detected as ED-frames. There was no significant difference in false ED-frame detection rate among the three vessels LAD (690, 43.07%), LCx (661, 46.26), and RCA (567, 41.27%).

**Figure 9:**
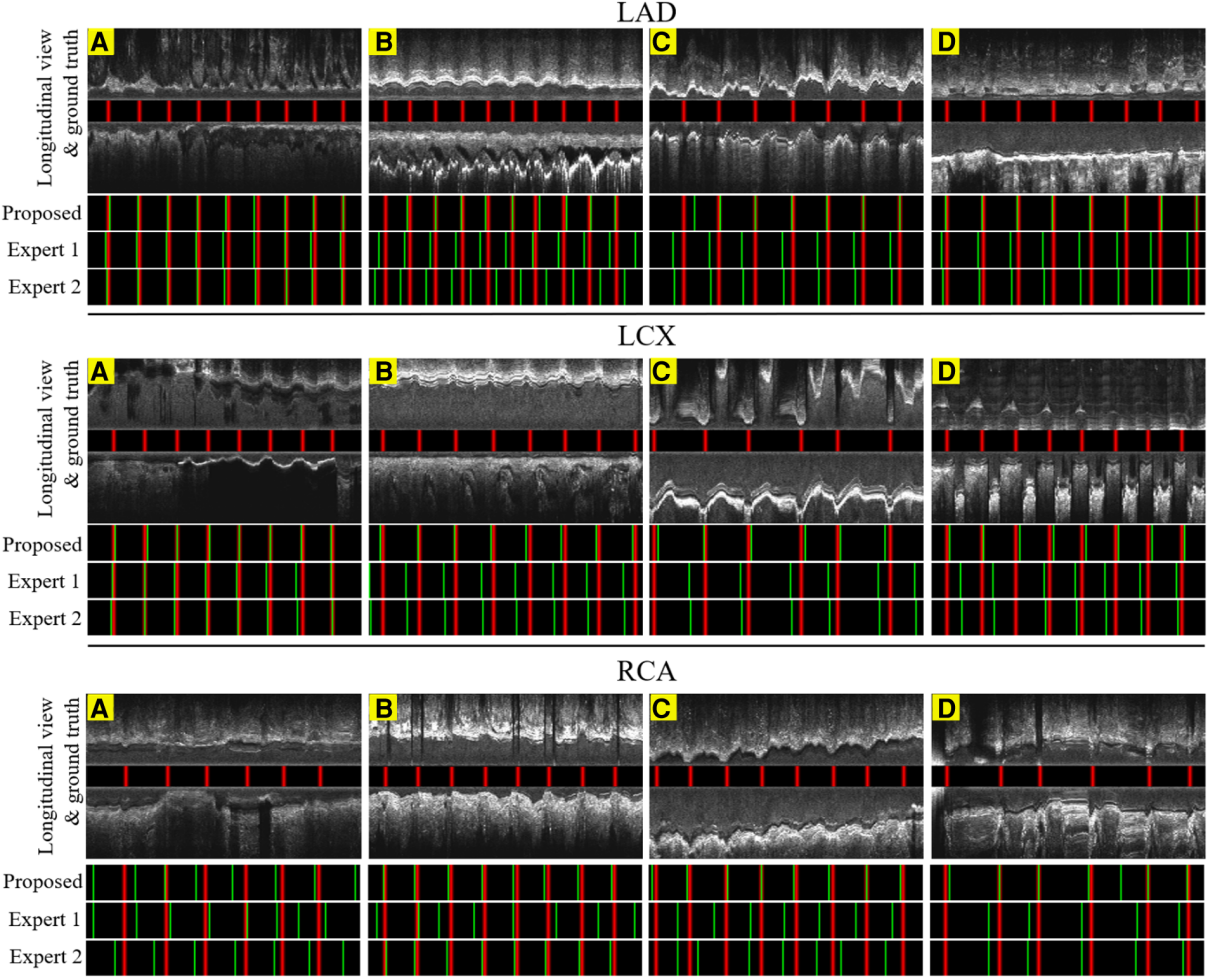
The result of the proposed gating method compared with the expert's predictions in 6.6 s or 200 frames. Red lines: ED-frames annotated by ECG; green lines: ED-frame predictions. The examples in LAD (**A**–**C**), LCX (**A**–**C**) and RCA (**B,C**) represent vessels with common motion patterns. In the cases LAD (**D**), LCX (**D**) and RCA (**A,D**), there is a smaller range of motion and artifacts making it harder for ED-frame detection.

Tables 3A,B presents the overall performance of two analysts, the LM methodology, and the proposed CARDIAN method regarding GoP recall, precision, and F1 score. CARDIAN outperforms the other methods, with a significant margin in all metrics. Table 4 further illustrates the performance for each coronary artery (LAD, LCX, and RCA). It is observed that expert analysts’ performance declines in RCA arteries in which vessel motion increases. In contrast, both the LM and CARDIAN methodologies demonstrate consistent performance across all coronary arteries. The results indicate that the CARDIAN methodology offers more robust performance across all coronary arteries, making it a more reliable choice for identifying ED frames in NIRS-IVUS sequences. This is particularly true when it comes to challenging cases with pronounced vessel motion, such as in the RCA. Some visual results are given in Figure 9.

**Table 3A:** Comparative performance evaluation of two experts the LM, and CARDIAN method in the entire dataset, based on a prediction tolerance of 66.7 ms (2 frames).

%	Expert 1	Expert 2	LM	BAF
GoP recall	22.72	29.02	20.11	69.94
GoP precision	21.19	27.20	19.02	56.46
F1 Score	21.93	28.08	19.55	62.48

**Table 3B:** The GoP recall, GoP precision, and GoP F1 score of ED-frame detection based on the CARDIAN method, the LM method (43), and visual annotations by two experts.

%	Vessel	Expert 1	Expert 2	LM	CARDIAN
GoP Recall	LAD	28.53	32.47	21.91	**71** **.** **87**
	LCX	28.16	34.86	19.33	**67**.**78**
	RCA	11.01	19.50	18.89	**69**.**93**
GoP Precision	LAD	26.12	29.94	20.38	**56**.**93**
	LCX	26.72	33.28	18.50	**53**.**74**
	RCA	10.29	18.28	17.99	**58**.**73**
GoP F1 score	LAD	27.27	31.15	21.12	**63**.**53**
	LCX	27.42	34.05	18.90	**59**.**95**
	RCA	10.64	18.87	18.43	**63**.**84**

The bold values indicate the highest scores for each coronary.

**Table 4:** The nearest prediction interval (in ms) of the two experts, the LM and the CARDIAN methodology.

	Expert 1		Expert 2		LM		Proposed	
	mean	std.	mean	std.	mean	std.	mean	std.
LCX	198.4	144.7	182.6	146.4	229.7	156.6	**82** **.** **7**	**101**.**1**
LAD	162.5	125.4	158.8	128	165.3	116.2	**65**.**2**	**86**.**5**
RCA	254.1	114.9	222.3	123	201.7	137.2	**82**.**1**	**83**.**5**
ALL	202.3	134	186	135.4	199	140.1	**76**.**9**	**92**.**4**

The values in bold represent the best performance in each coronary artery.

### Prediction interval evaluation

**Figure 10:**
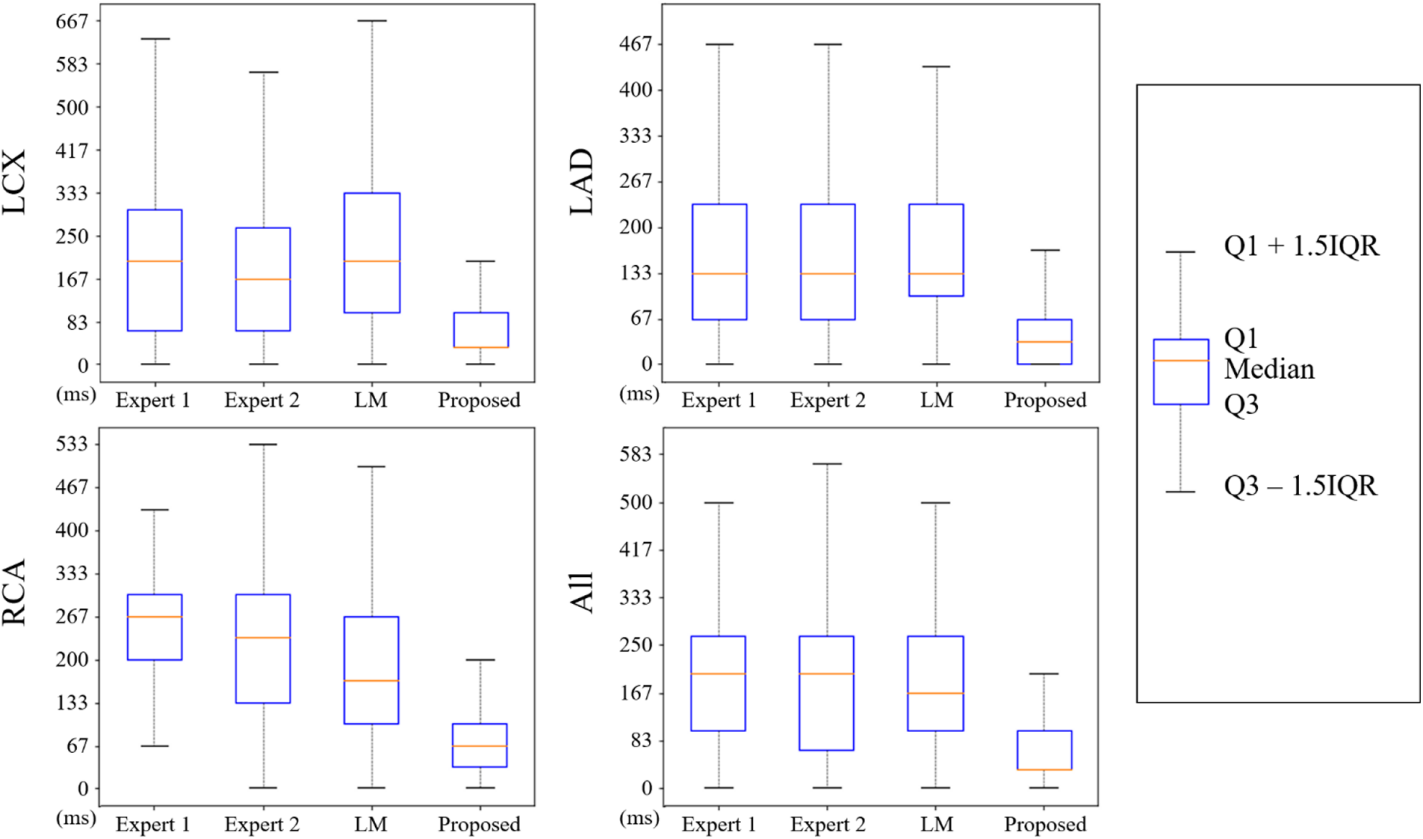
The distribution of nearest prediction interval between predicted ED-frames and ground truth, by expert 1, expert 2, LM, and the CARDIAN methodology.

The nearest prediction interval measurements for analysts 1 and 2, the LM and the CARDIAN methods across the three coronary arteries are shown in Table 4. It is apparent that the largest prediction interval values for Expert 1 and 2 are noted in the RCA where the vessel motion is larger, while for the LM method, the largest interval is noted in LCx. Conversely, the smallest nearest prediction interval values for both experts and the LM method are noted in the LAD. Compared to these results, the CARDIAN method demonstrated a significantly smaller nearest prediction interval and minimum variations across the three coronary arteries (Figure 10). For LCx, LAD, and RCA, the median of nearest prediction interval between the predicted ED-frame and the ground truth is 33.3, 33.3, and 66.6 ms, respectively.

## Discussion

This paper introduces a novel ED-frame detection approach, CARDIAN, that uses ECG-estimations as the gold standard for training and testing purposes. This approach takes advantage of specific features seen in IVUS sequences and the synchronous ECG tracings to accurately detect ED-frames, achieving superior performance compared to human experts and conventional image-based approaches (LM).

Over the last years, several computational approaches have been introduced for IVUS gating based on feature extraction and supervised methods, which assume that ED-frames are highly correlated to sudden changes in motion patterns. However, we have previously demonstrated (33) that extrema point detection cannot solely be used to reliably indicate the ED phase in an IVUS sequence. This should be attributed to the complex artery motion, which varies depending on the studied vessel, and imaging artifacts such as the presence of side branches or significant atherosclerotic lesions, making the visual identification of ED-frames a challenging task for humans and traditional image-analysis approaches (18).

The experiments in this study show that the proposed method CARDIAN can effectively achieve the real-time ED-frame detection task in IVUS sequences. The accurate detection of ED-frames is crucial for guiding interventional decisions, optimizing therapeutic interventions, and ensuring standardized volumetric analysis in IVUS studies. The CARDIAN framework integrates several dedicated computational methods to extract motion features and predict ED-frames, including dedicated motion encoders, a bidirectional attention recurrent network (BARNet), and a spatiotemporal rotation encoder. The motion encoders, including a temporal difference encoder and a spatiotemporal rotation encoder, extract frame-by-frame motion features corresponding to the phases of the cardiac cycle. The BARNet model predicts the likelihood of each frame being an ED-frame using a bidirectional recurrent network with an attention layer. The spatiotemporal rotation encoder captures the IVUS catheter's rotational movement with respect to the coronary artery. All these designs are obtained based on an in depth understanding of the true requirements of cardiovascular research, by analysing the unique properties of the data and task, and by putting together the most advanced computer vision and machine learning algorithms in a dedicated way for tackling the end diastolic frame detection challenge.

The deep learning solution of CARDIAN is shown to overcome the challenges and outperform both human expert analysts and conventional approaches, with results even superior to a GRU approach as previously described. This is attributed to the integration of motion, temporal, and spatiotemporal rotation encoders with a BARNet network. Unlike previous deep learning approaches that focus on feature extraction and shallow-learned representations, the CARDIAN approach takes advantage of the bidirectional attention mechanism of the BARNet to capture the temporal relationship between frames, essential for cardiac gating. The CARDIAN approach also considers the rotation of the IVUS catheter with respect to the coronary artery, an important motion feature previously overlooked. This highlights the novelty and superiority of the proposed CARDIAN approach for ED-frame detection in IVUS sequences.

Testing of the developed methodology against the ECG estimations underscores the potential but also the limitations of the CARDIAN methodology. We found that in contrast to the expert analysts and the LM method, the CARDIAN approach provides consistent results in all the 3 epicardial coronary arteries. More importantly, the performance of our approach is 2–3 times better than the conventional methodologies or manual screening. The performance of the CARDIAN approach was excellent in detecting the ED when a prediction tolerance of 100 ms was used, however, the superiority was still present when this cutoff was 66 ms.

A limitation of the present analysis is that it did not include patients with arrhythmias—such as atrial fibrillation or frequent ectopics—to evaluate the performance of the CARDIAN approach in these cases. Arrhythmia can affect the R-R interval, which is a crucial component of the CARDIAN approach for ED-frame detection. Despite this limitation, the proposed ED frame detection constitutes a key advance in IVUS image analysis and is expected to positively influence subsequent research. We have previously demonstrated that ED-frame-based volumetric IVUS analysis is more reproducible than conventional IVUS segmentation (44). These findings are important for longitudinal intravascular imaging-based studies assessing the implications of pharmacotherapies on plaque volume, as a more reproducible IVUS analysis is expected to reduce the number of vessels that should be included in these studies to demonstrate statistically significant changes in plaque burden (45, 46). Another limitation is undersized dataset based on which the experiments are performed, this is due to unavoidable constraints in the current practices, such as limited equipment availability, labor-intensive data collection, limited suitable patient cases, etc. These problems limit the number of samples available for training the model. We acknowledge and appreciate the support from InfraReDx, Inc. for this work. They have expressed interest in incorporating the developed CARDIAN approach into their system to accurately detect the ED-frame for more reproducible volumetric analysis. This collaboration further validates the potential impact and usability of the CARDIAN approach in real-world clinical and research settings.

## Conclusions

This study introduces a novel computational approach for real-time end-diastolic frame detection in intravascular ultrasound using bidirectional attention networks, CARDIAN, that is capable to accurately detect the EDs in the three coronary arteries. The proposed method operates in real-time and has superior performance to expert analysts and conventional LM methods. These advantages prove that this method is useful in clinical research and, in particular, in the analysis of large imaging datasets collected in longitudinal studies of coronary atherosclerosis.

## Data Availability

The raw data supporting the conclusions of this article is owned by the sponsor of the study and cannot be made available.
